# Pseudotumor and delayed recurrent dislocation after total hip arthroplasty with a modular femoral neck: A case report

**DOI:** 10.1097/MD.0000000000029056

**Published:** 2022-03-18

**Authors:** Zhe-Yu Huang, Shi-Cheng Wang, Hao-Jun Zhang, Long Shao, Zheng-Lin Di, Kun Tao

**Affiliations:** *The Department of Orthopedics Surgery, Ningbo No. 6 Hospital, Ningbo, Zhejiang, People’s Republic of China.*

**Keywords:** modular neck, pseudotumor, recurrent dislocation, total hip arthroplasty

## Abstract

**Rationale:**

Pseudotumor formation after hip arthroplasty is a rare complication that can occur not only at the head-neck junction but also at the modular neck-stem junction. Dislocation is a challenging and common complication of primary and revision total hip arthroplasty compared with other complications. Similarly, the association between pseudotumors and delayed recurrent dislocation remains unclear.

**Patient concerns:**

We report the case of a 73-year-old woman with pseudotumor formation after total hip arthroplasty combined with a modular femoral neck. A delayed recurrent dislocation occurred in this case. Approximately 4weeks after the first revision surgery, redislocation occurred.

**Diagnosis:**

The patient was eventually diagnosed with delayed recurrent artificial hip dislocation combined with a periprosthetic pseudotumor of the right hip.

**Interventions:**

During the first revision surgery, a thickened, indurated cyst measuring 8×3×8cm with a red-brown wall containing brown fluid was completely excised. A cemented stem, combined with a BIOLOX Forte ceramic head, was implanted. Approximately 4weeks after surgery, redislocation occurred, and we cemented an elevated rim liner on the acetabular component with a metal head.

**Outcomes:**

At the last follow-up, 49 months after revision surgery, the patient was asymptomatic with a Harris hip score of 90. The patient had a satisfactory prognosis after treatment.

**Lessons:**

The application of the modular-neck stem should be cautiously performed, particularly for modular prostheses containing different alloys. Pseudotumors and insufficient soft-tissue tension both contribute to hip instability, which may eventually lead to delayed repeated dislocation. In addition, femoral offset must be considered. Cement-liner technology may be used for aging patients who are less active. This case report, focusing on pseudotumors and delayed recurrent dislocations, aimed to identify factors that may support this diagnosis, which is easy to miss. Consequently, it can provide further details on the treatment process and alert orthopedic surgeons to this infrequent but important cause of delayed recurrent dislocation.

## 1. Introduction

More than 30 years have passed since the modular femoral neck was first used clinically.^[1]^ Modular-neck stem offers additional surgical options for patients with hip deformities that cannot be properly corrected using stems with modularity at only the head-neck junction. The modular neck stem exhibits some advantages, such as adjusting the femoral offset, femoral anteversion, leg length, and range of motion.^[2]^ However, the addition of a modular junction presents some disadvantages, including the possibility of dissociation,^[3]^ neck fracture,^[4]^ and fretting wear and corrosion at the neck-stem junction.^[5]^ This has caused some prostheses with modular necks to be recalled.^[6]^ Fretting wear and corrosion are major concerns, posing great risk from the modular neck-stem junction, which may precipitate metal hypersensitivity reactions, as seen in metal-on-metal total hip arthroplasty (THA).^[7]^ Terms used to describe metal hypersensitivity reactions include: adverse local tissue reactions, aseptic lymphocytic vasculitis-associated lesions, adverse reactions to metal debris, pseudotumors, and metallosis.^[8-10]^ The histology of pseudotumors exhibits features of metal hypersensitivity and metal wear reactions, with the presence of macrophages, lymphocytes, and cellular necrosis.^[11]^

Here, we report a case of pseudotumor formation after THA combined with a modular femoral neck. A delayed recurrent dislocation occurred in this case. A case of modular femoral neck with these 2 complications has not been previously reported.

## 2. Case presentation

A 73-year-old woman underwent primary right THA for traumatic avascular necrosis of the femoral head at our department for over 7years. She also presented with severe constant right-sided hip and groin pain and stiffness with difficulty walking over the previous year. Her medical history revealed that the patient had undergone internal fixation for a right femoral neck fracture at another institution 25 years previously. No other diseases (i.e., neuromuscular and cognitive disorders) were found, except for hypertension, which was well controlled with regular medication. A review of the previous surgical records revealed a history of cementless third-generation ceramic-on-ceramic THA through a posterolateral approach using a 48 mm lineage acetabular cup, size-4 PROFEMUR Z femoral stem, 39mm modular neck with 8° varus and a 28 mm Biolox Forte ceramic head with a 3.5mm head offset (all components from Wright Medical Technology, Arlington, TN). The prosthetic stem and modular neck were manufactured using titanium alloy and cobalt-chromium alloy (CoCr), respectively.

At the initial follow-up 1 month after surgery, she had no pain and a normal range of movement with no abductor weakness and was able to walk unaided. Radiographs showed good alignment with no evidence of hardware failure (Fig. 1A). At 39months postoperatively, the patient suffered from acute right hip pain with limited movement when sitting on a low stool. She immediately reported to our emergency department because she was unable to stand or bear weight on the right side. The affected leg also exhibited shortening with hip flexion, adduction, and internal rotation. Radiographs of right hip joint dislocation are shown in Figure 1B. Considering her medical history, physical signs, and imaging findings, posterior dislocation of the hip after arthroplasty was suspected. Closed reduction was successfully performed under general anesthesia. Afterward, she was advised to avoid weight-bearing and dangerous positions for 4 weeks and 3 months, respectively. At 41 months after arthroplasty, more than 2 months after the first dislocation, the patient experienced acute right hip pain with limited movement when turning the body. Symptoms, signs, and imaging findings were similar to those of the first dislocation. Hence, a closed reduction was conducted, and similar advice was reiterated (Fig. 1C). Right hip dislocation recurred 6 weeks after the second dislocation (Fig. 1D). After closed reduction, revision surgery was advised; however, she refused conservative treatment. Despite attempts at conservation, even staying in bed absolutely, a fourth dislocation occurred 2 months after the third (Fig. 1E). Closed reduction was conducted, followed by X-ray and computed tomography scans (Fig. 2) before revision. Magnetic resonance imaging was not performed because of metal artifacts. A computed tomography scan of the hip joint revealed a mass-like lesion behind the prosthetic stem with a small amount of calcified tissue, which appeared to be connected to the modular neck-stem junction (Fig. 2). Therefore, pseudotumors were suspected.

**Figure:**
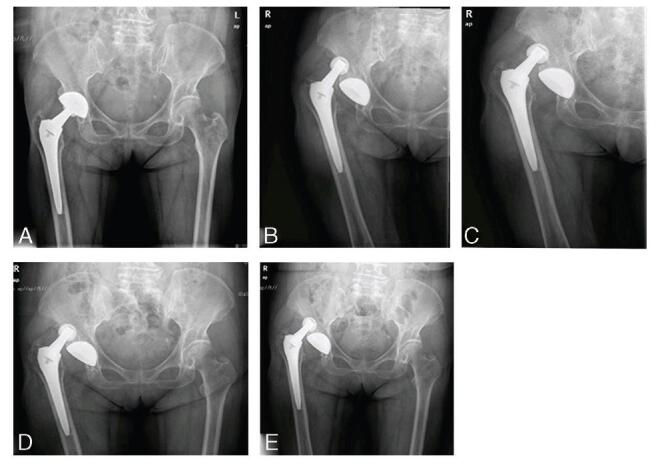
**Figure 1.** X-ray radiography of the artificial hip before the first revision. A, X-ray of the hip after primary hip arthroplasty. B, X-ray of the first dislocation of artificial hip. C, X-ray of the second dislocation of artificial hip. D, X-ray of the third dislocation of artificial hip. E, X-ray of the fourth dislocation of artificial hip.

**Figure:**
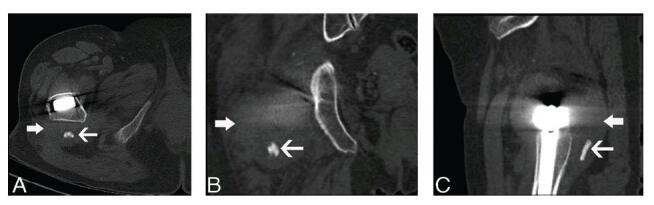
**Figure 2.** CT scan of the artificial hip before the first revision. A, Axial CT scan shows a mass-like lesion behind the prosthetic stem with a small amount of calcified tissue. B, Coronal CT scan shows a mass-like lesion behind the prosthetic stem with a small amount of calcified tissue. C, Sagittal CT scan shows a mass-like lesion behind the prosthetic stem with a small amount of calcified tissue. White thick arrow points to mass like lesion, and white thin arrow points to calcified tissue. CT = computed tomography.

The revision was performed using a previously described approach. The joint was filled with bloody fluid, with a thickened, indurated cyst measuring 8×3×8 cm and a red-brown wall posterolateral to the prosthetic stem and connected to the modular neck-stem junction (Fig. 3A). The cyst containing the brown fluid was completely excised. There was no extensive necrotic tissue or metallosis within the capsule. When revealing the modular neck-stem junction of the prosthesis, fretting, and black deposits were observed (Fig. 3B). The degree of fretting and black deposits in the head-neck taper were obviously lower than those in the modular neck-stem junction (Fig. 3B-E). The acetabular component was not revised because it was well fixated. Meanwhile, the stem component needed to be removed regardless of whether it was firmly fixed. Using flexible osteotomes and stem extractors, the stem was successfully removed, which was accompanied by a distal crack fracture of the femur. Three bands were evenly fixed on the proximal femoral shaft, followed insertion of an SPII cemented stem with a 28mm Biolox Forte ceramic head with -3.5mm head offset (all components from Link, Hamburg, Germany). After reduction and copious irrigation, the wound was closed in layers using absorbable sutures. The alignment of the prosthesis was confirmed immediately postoperatively, while a piece of cement was found at the lateral side of the distal femur, which may have oozed from the crack fracture (Fig. 4A). A few sutures at the distal end of the incision were removed, and a piece of cement was removed (Fig. 4B). The postoperative course was uneventful, and the patient was discharged after 5 days. Intraoperative bacterial cultures were negative, and postoperative histopathological examination revealed necrosis and lymphocytic infiltration (Fig. 5). The mass may also have been a pseudotumor originating from a metallosis.

**Figure:**
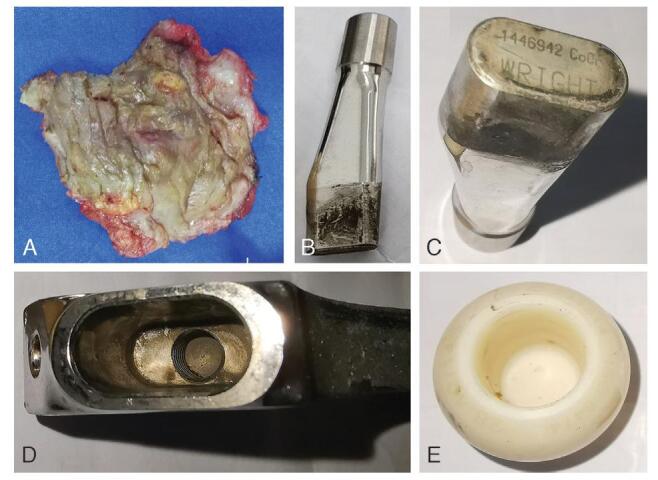
**Figure 3.** Intraoperative findings of the first revision. A, The excised pseudotumor shows a thickened, indurated cyst with a red-brown wall. B, The modular neck shows the degree of fretting and black deposit in the head-neck taper is obviously lesser compared with that in the modular neck-stem junction. C, The composition and serial number of the modular neck. D, The modular-neck stem shows the obvious black deposit in the junction. E, The BIOLOX Forte ceramic head shows the slight wear in the head-neck taper.

**Figure:**
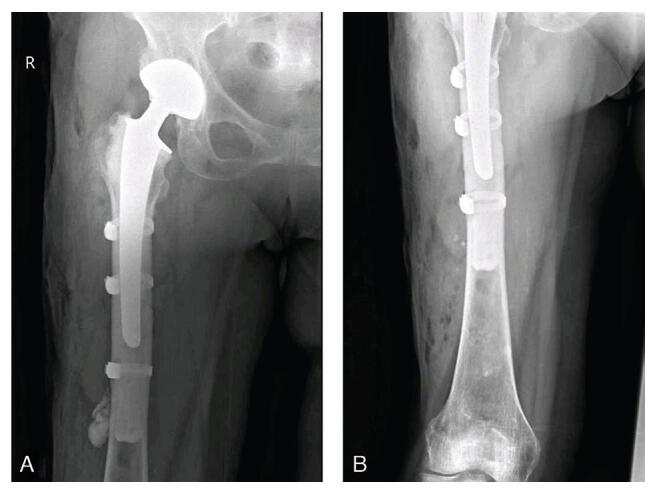
**Figure 4.** X-ray radiography of the hip immediately after the first revision. A, X-ray shows a piece of cement is found at the lateral of the distal femur. B, X-ray shows the piece of cement is removed.

**Figure:**
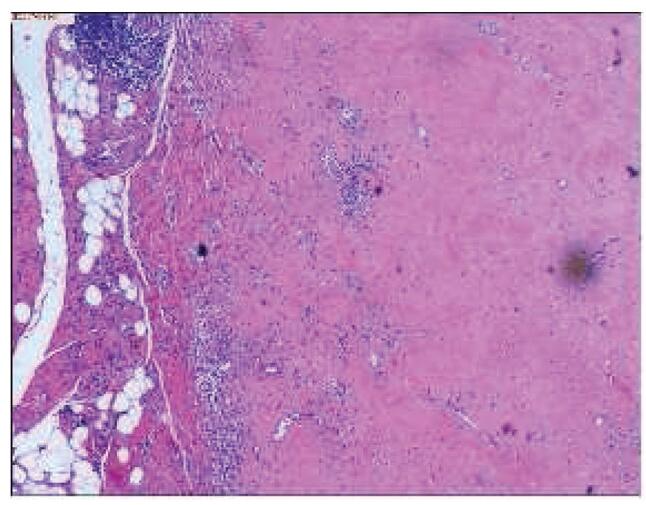
**Figure 5.** Histological image shows a band of lymphogranulomatous aggregate (hematoxylin and eosin, ×10 magnification).

At approximately 4 weeks after the revision surgery, dislocation occurred again when the patient turned her body (Fig. 6A). Femoral offset was considered the most likely cause after excluding other factors, and partial revision was immediately conducted. An elevated-rim liner was fixed on the acetabular component using cement; meanwhile, a 28 mm metal head with a -3.5mm head offset was applied instead of a long-neck head owing to the excessive force of reduction. The alignment of the prosthesis was confirmed immediately after the surgery (Fig. 6B). At the last follow-up after 49 months, the patient was asymptomatic with a Harris hip score of 90.

**Figure:**
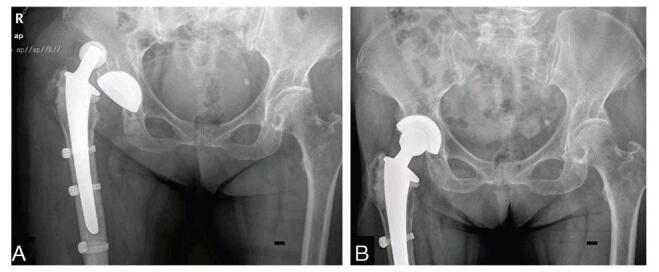
**Figure 6.** X-ray radiography of the hip at 4 weeks after the first revision. A, X-ray of redislocation of artificial hip. B, X-ray after the second revision. The scale bar indicates 10 mm.

## 3. Discussion

Modular neck stems have been introduced to anatomically, easily, and accurately reconstruct the hip joint. This provides greater flexibility in restoring hip version, offset, and leg length, independent of femoral stem positioning.^[12]^ Implant retrieval analysis has confirmed that crevice corrosion and/or fretting corrosion between the head and neck taper junction or modular neck and stem junction are common modes of failure.^[13,14]^ Different alloy combinations may yield different results. Duwelius et al^[15]^ reported no cases of revision or failure due to the femoral component in 634 patients treated with the M/L tapered hip prosthesis with Kinectiv (Zimmer, Warsaw, IN). Both the stem and modular neck were composed of titanium alloy (Ti6Al4 V). Meanwhile, Restrepo et al reported 41 hips (22.0%) that underwent revision because of neck-stem corrosion. The modular-neck stem of patients consisted of a titanium-molybdenum-zirconia-ferrite alloy stem and a CoCr modular neck.^[16]^ Serious adverse reactions have been observed in virtually all reported cases of cobalt-based alloys. Furthermore, an in vitro study showed that cobalt is more toxic to macrophages than other metals commonly used in implants.^[17]^ In this case, we reported usage of different alloys, which are a Ti6Al4V alloy stem in contact with a CoCr modular neck. In general, different alloys exhibit different elastic moduli. A Ti6Al4V alloy stem matching a CoCr modular neck may lead to greater fretting corrosion following local accumulation of cobalt ions, and an obvious black deposit in the modular neck-stem junction is also found during the operation. Consequently, a pseudotumor may have been formed. The toxicity of cobalt ions may damage and weaken soft tissues, especially abductors. A pseudotumor located posterolaterally to the prosthetic stem and connected to the modular neck-stem junction may affect hip joint movement, for example, abnormally increased mobility of the femur. As Saiz et al ^[18]^ reported, late first-time dislocations may indicate poor implant orientation because the normal biomechanics are disrupted. Therefore, physiological loading over time causes joint alterations, leading to instability, possible eccentric wear, and dislocation. In our patient, the pseudotumor and insufficient soft tissue tension contributed to hip instability, which eventually led to delayed repeated dislocation. It can be predicted that soft-tissue tension will worsen after pseudotumor resection because the space occupied by the pseudotumor is gone. Additionally, the femoral offset decreased after the first dislocation; therefore, the soft-tissue tension was insufficient. This may explain why dislocation occurred again four weeks after the first revision. Considering the patient’s age and activity level, a partial revision was performed. An elevated rim liner was fixed to the acetabular component using cement. The femoral offset increased by nearly 1 cm compared with the preoperative offset. Meanwhile, the insertion of an elevated rim liner further increased the hip joint stability.^[18]^ We call this method cement-liner technology, which is a durable option for appropriate patients.^[19]^ No further dislocation occurred, with good hip function.

In summary, pseudotumors are a rare complication following hip arthroplasty; however, its incidence may increase after placement of a modular-neck stem, especially 1 containing different alloys. This may be a factor leading to delayed dislocation, and orthopaedic surgeons should be aware of this infrequent but important cause. Cement-liner technology may be considered for aging patients who are less active.

## 4. Patient consent

Written informed consent was obtained from the patient for publication of this case report and accompanying images. A copy of the written consent form is available for review by the editor of this journal.

## Acknowledgments

The authors thank Editage for assisting in the preparation of this manuscript.

## Author contributions

**Data curation:** Zhe-Yu Huang, Shi-Cheng Wang.

**Formal analysis:** Zhe-Yu Huang, Hao-Jun Zhang, Zheng-Lin Di, and Kun Tao.

**Investigation:** Zhe-Yu Huang, Hao-Jun Zhang, Long Shao.

**Methodology:** Zhe-Yu Huang.

**Project administration:** Kun Tao.

**Writing** - **original draft:** Zhe-Yu Huang.

**Writing** - **review & editing:** Zheng-Lin Di, Kun Tao.
